# Panniculitis in Anti-MDA-5 Positive Juvenile Dermatomyositis: A Case Report

**DOI:** 10.31729/jnma.8874

**Published:** 2025-01-31

**Authors:** Semra Ayduran, Gulsah Kilbas, Saadet Nilay Tigrak, Selcuk Yuksel, Erdem Comut, Serkan Turkucar

**Affiliations:** 1 Department of Pediatric Rheumatology, Pamukkale University, Denizli, Turkiye; 2 Department of Pediatric Rheumatology, Canakkale Onsekiz Mart University, Canakkale, Turkiye; 3 Department of Pathology, Pamukkale University, Pathology, Denizli, Turkiye

**Keywords:** *anti MDA-5*, *dermatomyositis*, *panniculitis*

## Abstract

Panniculitis is a rare clinical finding in dermatomyositis. There are few reported cases in the medical literature. In this report, we describe a 17-year-old male patient with anti-MDA5 positive hypomyopathic dermatomyositis who, eight months after diagnosis, presented with indurated nodules on the right forearm and right thigh despite methotrexate and monthly intravenous immunoglobulin treatment. A skin biopsy revealed lobular panniculitis with lymphocytic infiltrate. His lesions were successfully controlled with hydroxychloroquine and azathioprine. This article presents a case regarding the rarity of panniculitis in juvenile dermatomyositis and its treatment strategy.

## INTRODUCTION

Juvenile Dermatomyositis (JDM) is a chronic autoimmune condition affecting the skin and muscles, often linked to capillary vasculopathy. Hypomyopathic dermatomyositis describes patients with typical JDM skin symptoms but no clinical muscle involvement, although subclinical myositis may be indicated by elevated muscle enzymes, electromyography (EMG), magnetic resonance imaging (MRI), or muscle biopsy.^1^

Panniculitis is characterized by painful, erythematous nodules or plaques caused by inflammation of subcutaneous tissues. It is exceptionally uncommon in patients with JDM.^2,3^ This report presents a case of hypomyopathic dermatomyositis with the rare subcutaneous manifestation of panniculitis and anti-MDA5 positivity.

## CASE

**Figure 1:**
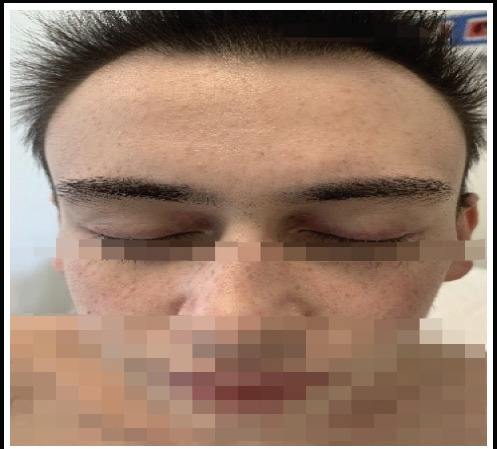
Heliotrope rash mentioned in the case.

**Figure 2:**
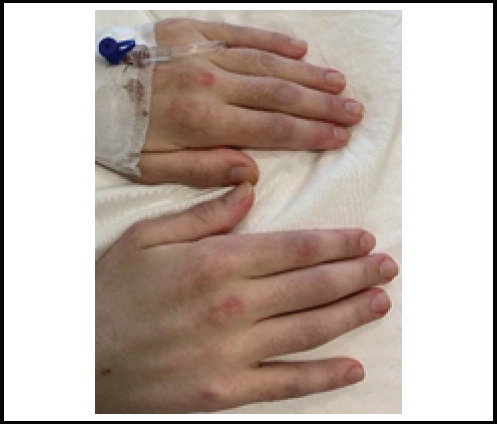
Gottron's papules mentioned in the case.

A 17-year-old male was referred to our clinic with a 6-month history of significant weight loss, joint pain, and a skin rash. Physical examination revealed scaly, erythematous rashes on the scalp and eyelids, indicative of a heliotrope rash (Figure 1). Gottron papules (Figure 2) were observed on the metacarpophalangeal joints, along with red papules on the extensor surfaces of the elbows. The patient demonstrated full muscle strength (5/5) in all extremities.

Laboratory tests showed anemia (Hb: 10.1 g/dL), lymphopenia (absolute lymphocyte count: 700 K/uL), an elevated sedimentation rate (70 mm/h), increased aspartate aminotransferase (AST) (75 IU/L), and elevated troponin levels (23.7 ng/L). Creatine kinase (CK) (103 U/L) was within normal limits. There were no signs of hematological malignancy in the peripheral blood smear. Anti-MDA5 myositis specific antibody was detected with +3 positive high titer. Muscle Magnetic Resonance Imaging (MRI) of the thighs showed increased signals indicating myositis, while EMG revealed low-amplitude polyphasic motor unit potentials, consistent with myopathic involvement. A skin biopsy from the right elbow confirmed capillary dilation and perivascular lymphoid infiltration, consistent with JDM.

Although there were no clinical signs of muscle involvement, the patient was diagnosed as hypomyopathic juvenile dermatomyositis with typical skin manifestations, elevated AST levels and the findings of subclinical myositis in electromyogram (EMG) and MRI examinations. High-resolution chest computerized tomography (CT) and respiratory function tests, carbon monoxide diffusion tests were normal, as were echocardiographic evaluations.

Oral methylprednisolone was administered at 2 mg/kg daily after a three-day intravenous pulse of 30 mg/kg per day. During the tapering process, methotrexate (MTX) (15 mg/m^2^ per week) and monthly intravenous immunoglobulin (IVIG) (2 g/kg) treatments were initiated.

**Figure 3:**
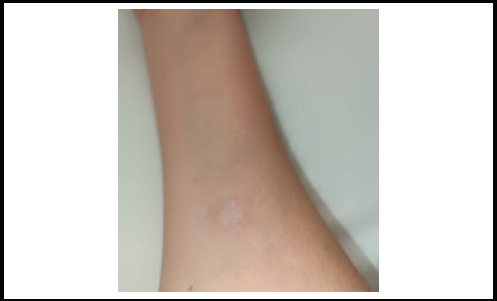
Panniculitis on the forearm mentioned in the case.

**Figure 4:**
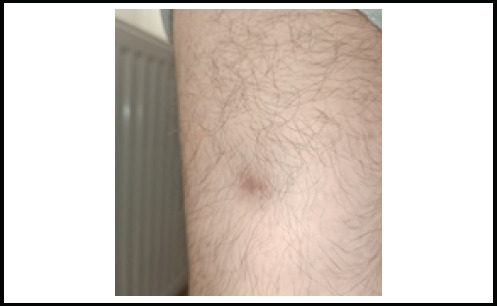
Panniculitis on the lateral thigh mentioned in the case.

**Figure 5:**
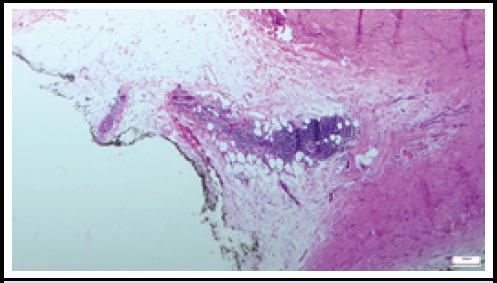
Panniculitis characterized by mononuclear cell infiltration in subcutaneous adipose tissue (H&E, x50 maginification).

In the eighth month of the disease, painless indurated plaques developed on the right forearm and later on the right thigh, despite improvements in other skin symptoms (Figures 3, 4). An excisional biopsy showed intense mononuclear cell infiltration in the perivascular area of the subcutaneous tissue and lymphocyte infiltration in the vessel walls, along with minimal infiltration and mucin accumulation in the dermis, consistent with panniculitis. (Figure 5). Therefore, oral methylprednisolone, which had been discontinued in the seventh month, was reintroduced, while MTX was switched to azathioprine and hydroxychloroquine. The patient continued receiving IVIG. Due to positive anti-MDA5 antibodies, close monitoring with a thoracic CT scan and pulmonary function tests was implemented to evaluate the risk of interstitial lung disease. The patient is under strict follow up and the subcutaneous lesions remained stable.

## DISCUSSION

Hypomyopathic JDM is a rare childhood disease that is challenging to diagnose due to the absence of muscle symptoms alongside skin manifestations. Panniculitis, an inflammation of the subcutaneous fat, is an uncommon skin manifestation compared to typical JDM rashes like Gottron's papules and the heliotrope rash. A case of hypomyopathic JDM with panniculitis is discussed, which developed despite treatment with MTX and monthly IVIG. The condition was successfully managed with hydroxychloroquine and azathioprine, underscoring the complexities of treating rare dermatomyositis variants in children.

In clinical terms, panniculitis in JDM manifests as tender, indurated, erythematous, subcutaneous nodules, occasionally accompanied by multifocal lipoatrophy, particularly in younger cases.^4^ Studies show that in most patients, panniculitis often develops after other symptoms of JDM, usually taking about 2 to 3 years from onset to diagnosis.^5^ In our case, panniculitis appeared silently with lipoatrophy eight months after diagnosis, unlike the typical painful presentation.

Recent literature reports nearly 60 cases of panniculitis linked to adult dermatomyositis and fewer than 10 cases in JDM.^5,6,7^ While clinical cases are rare, histopathological evidence is more common, with about 9% of JDM biopsy samples showing panniculitis. MRI has also revealed subcutaneous edema in the thighs and hips of many JDM patients.^5^ Histopathological findings in JDM associated panniculitis include lobular panniculitis, hyalinized fat necrosis, paraseptal lymphoid aggregates, and perivascular mononuclear cell infiltration with lymphocytic vasculitis, similar to our case.^6^ The simultaneous occurrence of panniculitis, myositis, and corresponding histological changes in skeletal muscle and subcutaneous fat suggests a potential common pathogenesis. Angiopathy may indicate a shared factor in the development of both conditions.^8^ However, the precise pathogenesis of panniculitis in dermatomyositis remains unclear.

Recent studies link anti-MDA5 antibodies to JDM, rapidly progressive interstitial lung disease, and joint involvement. Common features include skin ulcerations, palmar papules, mechanic's hands, alopecia, and oral ulcers.^9^ Fewer than 10 adult cases of dermatomyositis with panniculitis and positive anti-MDA5 antibodies have been reported, and no pediatric cases have been found.^2^ A study by Labrador-Horrillo et al. found that panniculitis occurs significantly more in adult-DM patients with anti-MDA5 antibodies (35.7%) than in those without (12.6%), alongside a complication rate of 35.7%.^10^ There is also a positive correlation between anti-MDA5 antibody levels and disease activity in JDM. Early aggressive treatment can improve lung lesions and panniculitis, leading to decreased anti-MDA5 antibody titers.^2^

There is no specific treatment protocol for panniculitis in dermatomyositis, but systemic corticosteroids are the main treatment. If resistant, options include cDMARDs and immunosuppressants like MTX, hydroxychloroquine, azathioprine, cyclosporine, mycophenolate mofetil, and cyclophosphamide. In further cases, bDMARDs such as etanercept, rituximab, and IVIG may be considered.^2^

The coexistence of anti-MDA5 positive dermatomyositis and panniculitis is very rare in childhood period, and this is the first JDM case in the literature to the best of our knowledge. The relationship between the presence of myositis-specific antibodies, such as anti-MDA5, and the panniculitis is not clarified enough, which highlighting the necessity for extensive studies. In conclusion, considering panniculitis among the diagnostic features of JDM could be valuable for a more comprehensive understanding of the disease.
